# Large Lateral Photovoltaic Effect in MoS_2_/GaAs Heterojunction

**DOI:** 10.1186/s11671-017-2334-z

**Published:** 2017-10-10

**Authors:** Lanzhong Hao, Yunjie Liu, Zhide Han, Zhijie Xu, Jun Zhu

**Affiliations:** 1 College of Science, China University of Petroleum, Qingdao, 266580 Shandong People’s Republic of China; 2 State Key Laboratory of Electronic Thin Films and Integrated Devices, University of Electronic Science and Technology of China, Chengdu, 610054 People’s Republic of China

**Keywords:** MoS_2_, GaAs, Photovoltaic, Heterojunction, Interface

## Abstract

Molybdenum disulfide (MoS_2_) nanoscaled films are deposited on GaAs substrates via magnetron sputtering technique, and MoS_2_/GaAs heterojunctions are fabricated. The lateral photovoltaic effect (LPE) of the fabricated MoS_2_/GaAs heterojunctions is investigated. The results show that a large LPE can be obtained in the MoS_2_/*n*-GaAs heterojunction. The LPE exhibits a linear dependence on the position of the laser illumination and the considerably high sensitivity of 416.4 mV mm^−^^1^. This sensitivity is much larger than the values in other reported MoS_2_-based devices. Comparatively, the LPE in the MoS_2_/*p*-GaAs heterojunction is much weaker. The mechanisms to the LPE are unveiled by constructing the energy-band alignment of the MoS_2_/GaAs heterojunctions. The excellent LPE characteristics make MoS_2_ films combined with GaAs semiconductors promising candidates for the application of high-performance position-sensitive detectors.

**Electronic supplementary material:**

The online version of this article (10.1186/s11671-017-2334-z) contains supplementary material, which is available to authorized users.

## Background

Due to its excellent properties, molybdenum disulfide (MoS_2_) is being investigated as one typical kind of two-dimensional materials to develop next-generation microelectronic devices and optoelectronic devices [1–5]. Unlike graphene, MoS_2_ has obvious band gap and its band gap decreases with increasing layer numbers [6]. The presence of the obvious band gap allows the fabrication of the MoS_2_ transistors with an on/off ratio exceeding 10^8^ and the photodetectors with high responsivity [7, 8]. Recently, MoS_2_ combined with other semiconductors has attracted much interest, such as GaAs, Si, and GaN [9–13]. These designed heterostructures supply feasible technical route for MoS_2_-based materials to develop practically applicable optoelectronic devices. Among all these bulk semiconductors, GaAs has a suitable direct band gap of ~ 1.42 eV and high electron mobility (~ 8000 cm^2^ V^−1^ s^−1^). Lin et al. fabricated MoS_2_/GaAs solar cells with a power conversion efficiency over 9.03% [9]. Further, Xu et al. reported a MoS_2_/GaAs self-driven photodetector with the extremely high detectivity of 3.5 × 10^13^ Jones [10]. In previous reports, the studies on MoS_2_/GaAs heterostructures have been mainly focused on the application in the area of solar cells and photodetectors. However, the MoS_2_/GaAs as a position-sensitive detector (PSD) based on the lateral photovoltaic effect (LPE) has been reported rarely. Different from the ordinary longitudinal photovoltaic effect, the LPE originates from the lateral diffusion and recombination of the photon-generated carriers in the inversion layer at the interface [14–18]. In the LPE effect, a lateral photovoltage (LPV) can be obtained and it changes linearly with the laser spot position on the active region of the device surface. These characteristics make LPE very useful in developing high-performance PSDs and have been studied widely in the area of robotics, biomedical applications, process control, position information systems, and so on.

In this work, MoS_2_ thin films with different thickness are deposited on the surface of *n*-/*p*-GaAs substrates via magnetron sputtering technique. A large LPE is observed in the fabricated MoS_2_/*n*-GaAs heterojunction, and the sensitivity reached 416.4 mV mm^−1^. Our results further show that the LPE exhibits obvious dependence on the carrier types of the GaAs substrates and the thickness of the MoS_2_ films. Through the construction of the energy-band alignment at the interface, the mechanisms to the LPE in the devices are proposed.

## Methods

MoS_2_ thin films were deposited on (100)-oriented GaAs substrates using the DC magnetron sputtering technique. The MoS_2_ powders (purity, ~ 99%) were cold-pressed into a disk under the pressure of 20.0 MPa. The as-fabricated disk (Φ60.0 mm × 4.5 mm) was used as the target during sputtering. The *n-*/*p*-GaAs substrates were used in our experiments, respectively. Before the deposition, the substrates were ultrasonically cleaned in sequence by alcohol, acetone, and de-ionized water. Subsequently, MoS_2_ thin films with different thickness (*d*_MoS2_ = ~ 10, 30, 50, 90 nm) were grown on the GaAs substrates at the temperature of 400 °C, respectively. During the deposition, the working pressure and power were kept at 1.0 Pa and 10.0 W, respectively. As a reference, MoS_2_ thin films were also deposited on intrinsic GaAs (*i*-GaAs) substrates under the same condition. Finally, about 300-μm In pads with a diameter of 0.5 mm as electrodes were pressed on the MoS_2_ film.

The MoS_2_ films were characterized using Raman spectroscopy (HORIBA, HR800) with the excitation wavelength of 488 nm. The surface of the sample was scanned by an atomic force microscope (AFM). X-ray photoemission spectroscopy (XPS) was performed by a Kratos Axis ULTRA spectrometer with a monochromatic Al Kα X-ray source. The deposition rate was obtained by the thickness from the cross-sectional scanning electron microscope (SEM) (Additional file 1: Figure S1) and the deposition time, then each film thickness was determined by the deposition rate and each deposition time. The transmission spectra were measured by Shimadzu UV-3150 spectrophotometer. Ultraviolet photoelectron spectroscopy (UPS) was performed using an unfiltered He-I (21.22 eV) gas discharge lamp. LPVs were measured using a Keithley 2000 voltmeter and three-dimensional electric motorized stage with a laser of 650-nm wavelength as the illumination source. The current-voltage (*I*-*V*) curves were measured with a Keithley 2400 SourceMeter.

## Results and Discussion

**Fig. 1:**
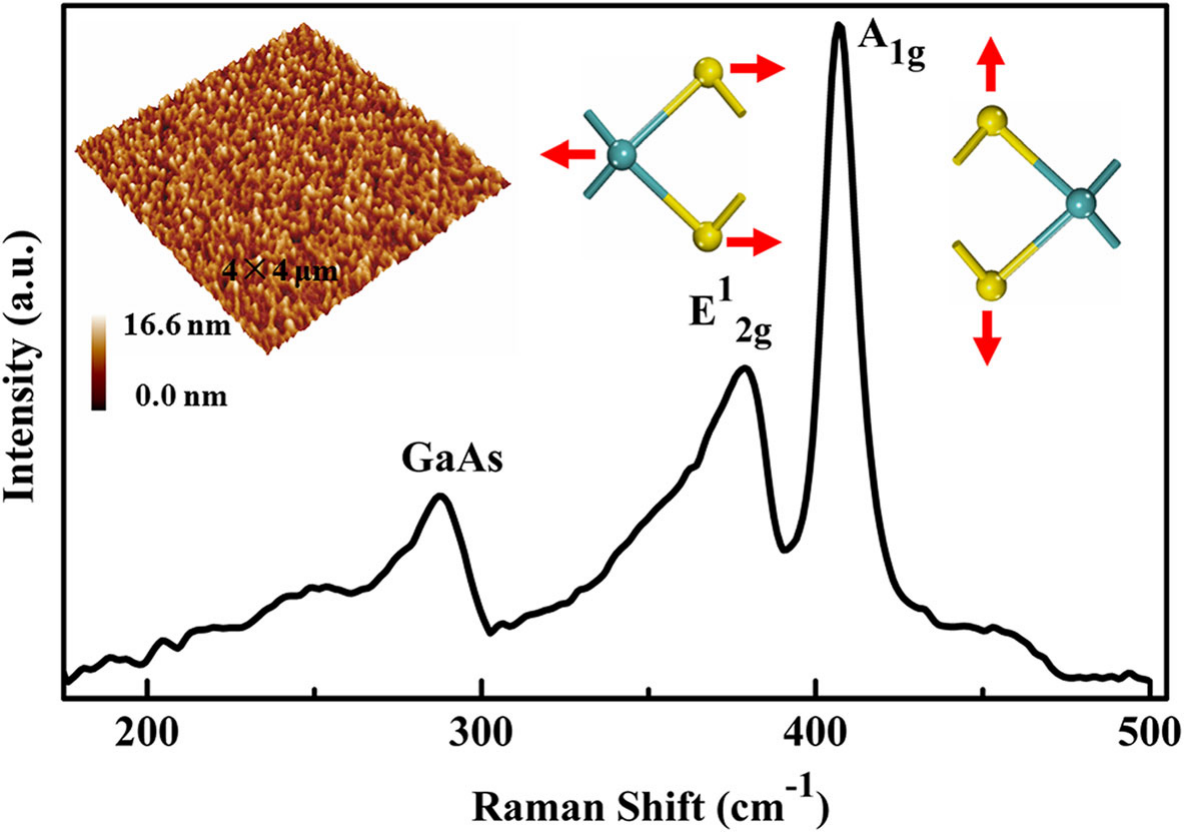
Raman spectrum of the MoS_2_ film on GaAs. The right two insets show the schematic illustrations of the oscillating mode of E^1^_2g_ and A_1g_, respectively. Atom color code: light blue-green, Mo; yellow, S. The left inset shows the surface morphology image of the as-grown MoS_2_ films

Figure 1 shows the Raman spectrum of the MoS_2_ film on the GaAs substrate. Besides the peak of the GaAs substrate at ~ 287.1 cm^−1^, two characteristic MoS_2_ Raman peaks can be seen, the A_1g_ mode at ∼ 406.7 cm^−1^ and E^1^_2g_ mode at ∼ 378.9 cm^−1^. The right two insets show the illustration of the atomic vibration in MoS_2_. The A_1g_ mode corresponds to the S atoms oscillating in antiphase along the out-of-plane direction, and the E^1^_2g_ mode corresponds to the S and Mo atoms oscillating in antiphase parallel to the crystal plane. As shown in the figure, the Raman peak corresponding to the A_1g_ mode is preferentially excited for the film. According to our measurements, the intensity ratio of A_1g_/E^1^_2g_ is about 2.1. These Raman characteristics are similar with other reported results about MoS_2_ thin films [19]. The left inset shows an AFM topographic image of the 40-nm MoS_2_ film grown on the GaAs substrate. From the figure, we can see that the surface of the film is composed of dense cone-like grains. According to the measurements, the root-mean-square (RMS) roughness of the film is about 1.7 nm, and the average size of grains is about 76.3 nm in diameter. These grains on the surface could decrease the surface reflection to the external light and enhance the light absorption of the fabricated device.

**Fig. 2:**
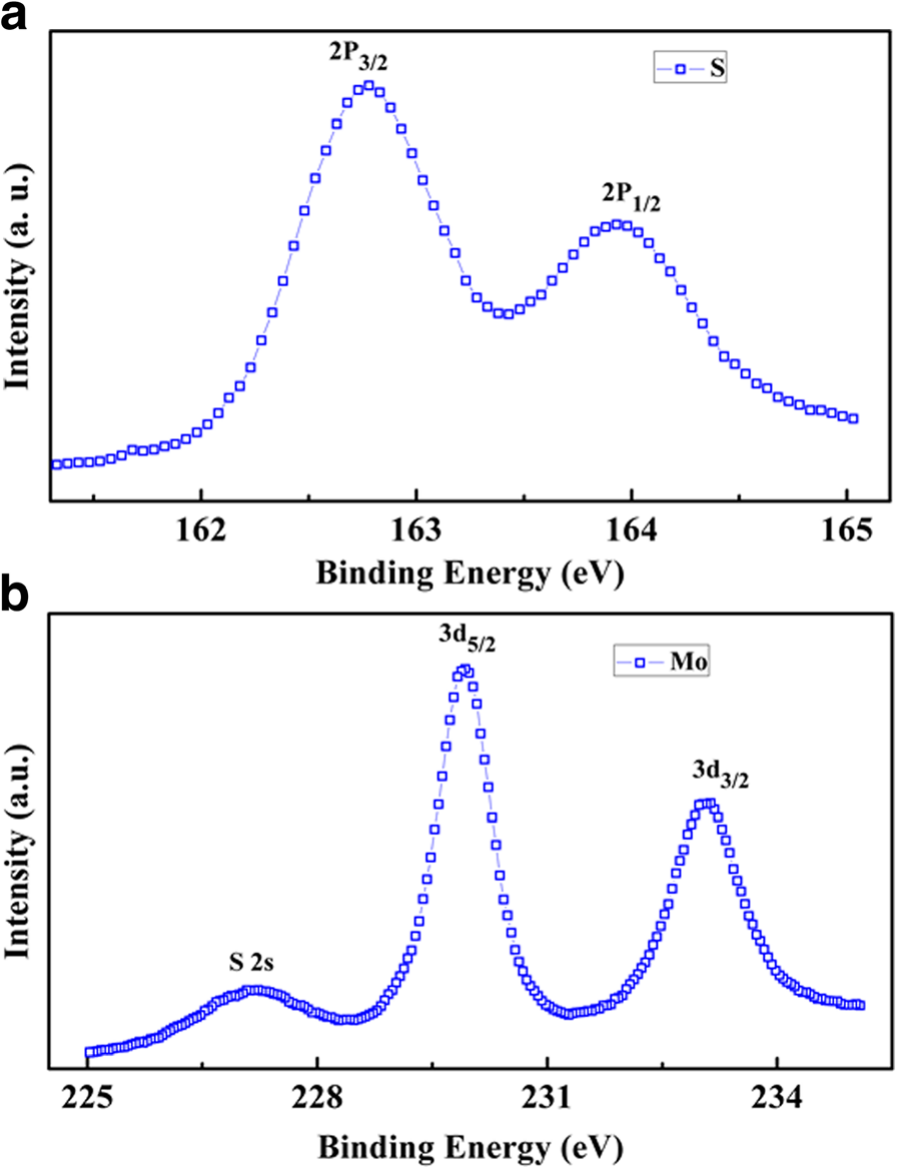
XPS spectra of the MoS_2_ film for **a** Mo and **b** S elements, respectively

Figure 2 shows the XPS spectra of the MoS_2_ film. As shown in Fig. 2a, the peaks at 229.3 and 232.5 eV are related to the Mo 3d_5/2_ and Mo 3d_3/2_ orbitals, respectively. As shown in Fig. 2b, S 2p_3/2_ and S 2p_1/2_ orbitals of divalent sulfide ions (S^2−^) are observed at 162.2 and 163.3 eV, respectively. The results are in good agreement with the reported values for the MoS_2_ crystal [17, 18].

**Fig. 3:**
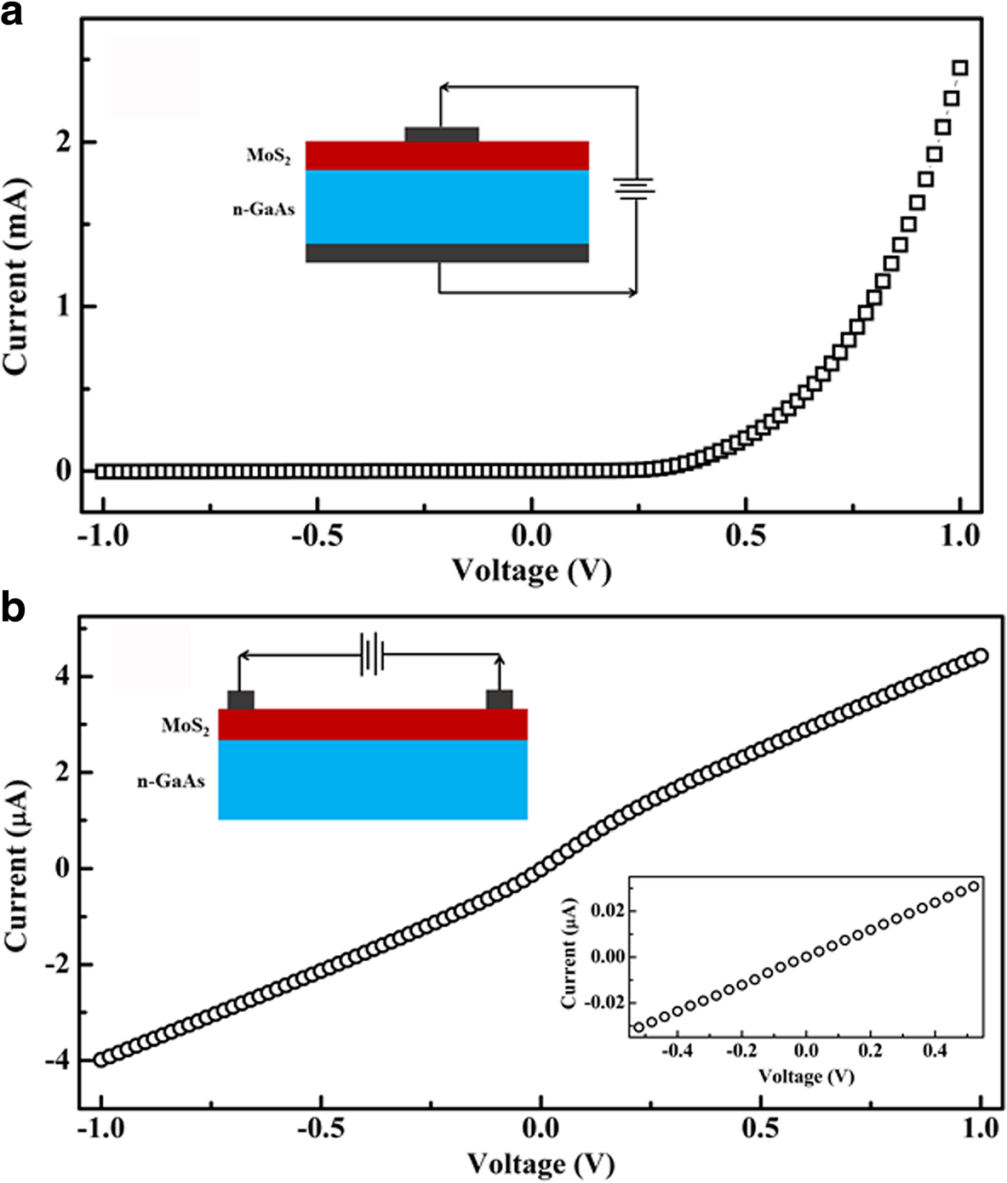
**a** Longitudinal *I*-*V* curve of the fabricated MoS_2_/*n*-GaAs heterojunctions. The inset shows the schematic circuit for the longitudinal measurements. **b** Transverse *I*-*V* curves of the fabricated MoS_2_/*n*-GaAs heterojunctions. The top inset shows the schematic circuit for the transverse measurements. The bottom inset shows the *I*-*V* curves of the MoS_2_ films on the intrinsic GaAs substrate

Figure 3a shows the longitudinal *I*-*V* curve of the fabricated MoS_2_/*n*-GaAs heterojunctions. Two In electrodes with the diameter of about 0.5 mm were pressed on the surface of the MoS_2_ film and the backside of the GaAs, respectively. The inset shows the schematic circuit for the longitudinal measurements. The forward voltage is defined as a positive bias voltage applied on the top In electrode. As shown in the figure, the fabricated MoS_2_/*n*-GaAs heterojunction shows obvious rectifying behaviour. The rectifying ratio (*I*_*+*_/*I*_*−*_) measured at ± 1.0 V is about 520. In our experiments, both In/MoS_2_ and In/GaAs belong to ohmic contacts and their *I*-*V* curves are almost linear. Thus, the rectifying *I*-*V* characteristic in the heterojunction is mainly originated from the MoS_2_/GaAs contact. Figure 3b shows the transverse *I*-*V* curve of the fabricated MoS_2_/*n*-GaAs heterojunctions. Two In electrodes with the diameter of about 0.5 mm were pressed on the surface of the MoS_2_ film. The top inset shows the schematic circuit for the transverse measurements. From the figure, the *I*-*V* curve shows slightly nonlinear increase of the currents with increasing voltages. This indicates that an inversion layer at the MoS_2_/*n*-GaAs interface is formed [18]. The bottom inset shows the *I*-*V* curves of the single MoS_2_ films on the intrinsic GaAs substrate. From the figure, an almost linear *I*-*V* curve can be seen, further indicating the ohmic nature of the In/MoS_2_ contact. At the voltage of + 0.5 V, the current of the single MoS_2_ is about 3.1 × 10^−2^ μA, much smaller than the value in the MoS_2_/*n*-GaAs, about 2.3 μA. Thus, compared to the MoS_2_ film, the inversion layer at the MoS_2_/*n*-GaAs interface supplies a path with a much lower resistivity for carrier transport during the transverse measurements of the MoS_2_/*n*-GaAs heterojunction.

**Fig. 4:**
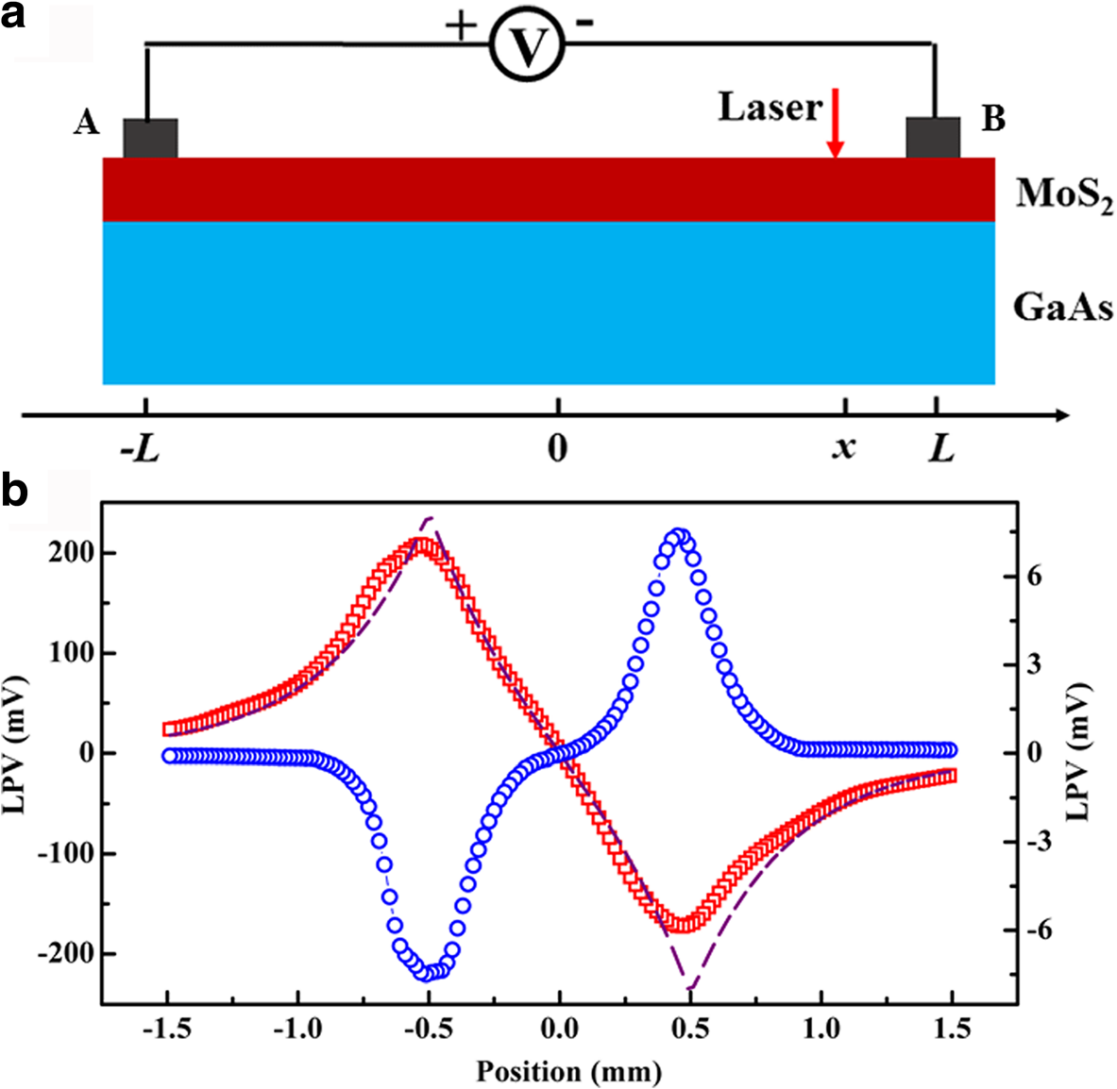
**a** Schematic circuit for the measurement of the LPE. **b** LPE curves of the MoS_2_/*n*-GaAs and MoS_2_/*p*-GaAs heterojunctions, respectively

Figure 4a shows the schematic circuit for the measurement of the LPE of the fabricated MoS_2_/GaAs heterojunction. Two In electrodes with the diameter of 0.5 mm are pressed on the surface of the MoS_2_ film to perform the measurements of the LPE. The distance (2*L*) between the electrodes is ~ 1.0 mm. During our measurements, electrodes A and B were connected to the positive and negative probes of a Keithley 2000 voltmeter, respectively. Figure 4b shows the LPE curves of the MoS_2_/*n*-GaAs and MoS_2_/*p*-GaAs heterojunctions, respectively. The thickness of the MoS_2_ films is ~ 30.0 nm. When the surface of the MoS_2_ film is partially illuminated by a laser spot with the diameter of about 0.1 mm, a large LPE can be observed in the MoS_2_/*n*-GaAs heterojunction. As shown in the figure, the LPE shows an approximately linear dependence on the laser spot position when the laser spot moves between electrodes A and B on the MoS_2_ surface. From the figure, we can see that the LPV depends on the position of the laser spot. This can be fitted with the diffusion theory [16],

$$ \mathrm{LPV}={K}_0\Big[\exp \left(-\frac{\left|L-x\right|}{d}\right)-\exp \left(-\frac{\left|L+x\right|}{d}\right) $$

where *K*_0_, 2*L*, *d*, and *x* represent a proportionality coefficient, the distance between two electrodes, the carrier diffusion length, and the laser spot position, respectively. The well-fitted results in the figure clearly suggest that the LPE in the MoS_2_/*n*-GaAs heterojunction arises from the lateral diffuse flow and recombination of the excited carriers away from the laser position. As shown in the figure, the LPV value is zero when the light spot is at the centre between two electrodes, which can be attributed to the diffusion symmetry of the carriers. When the light position is close to the A electrode, the LPV is positive and vice versa. This indicates that the LPE in the MoS_2_/*n*-GaAs heterojunction is caused by the hole-type photoexcited carriers. The maximum LPV is obtained when the laser illumination is closest to the electrodes. According to our measurements, the maximum lateral photovoltage (LPV_max_) is about 208.2 mV in the linear region of the MoS_2_/*n*-GaAs heterojunction. Comparatively, the LPV of the MoS_2_/*p*-GaAs heterojunction is much smaller and its LPV_max_ is only 7.3 mV, as shown in the figure. From the figure, we can see that the LPE of the MoS_2_/*p*-GaAs heterojunction is determined by the electron-type photoexcited carriers. Additionally, nonlinear LPE characteristics of the MoS_2_/*p*-GaAs heterojunction can be seen from the figure when the laser spot moves between the A and B electrodes.

**Fig. 5:**
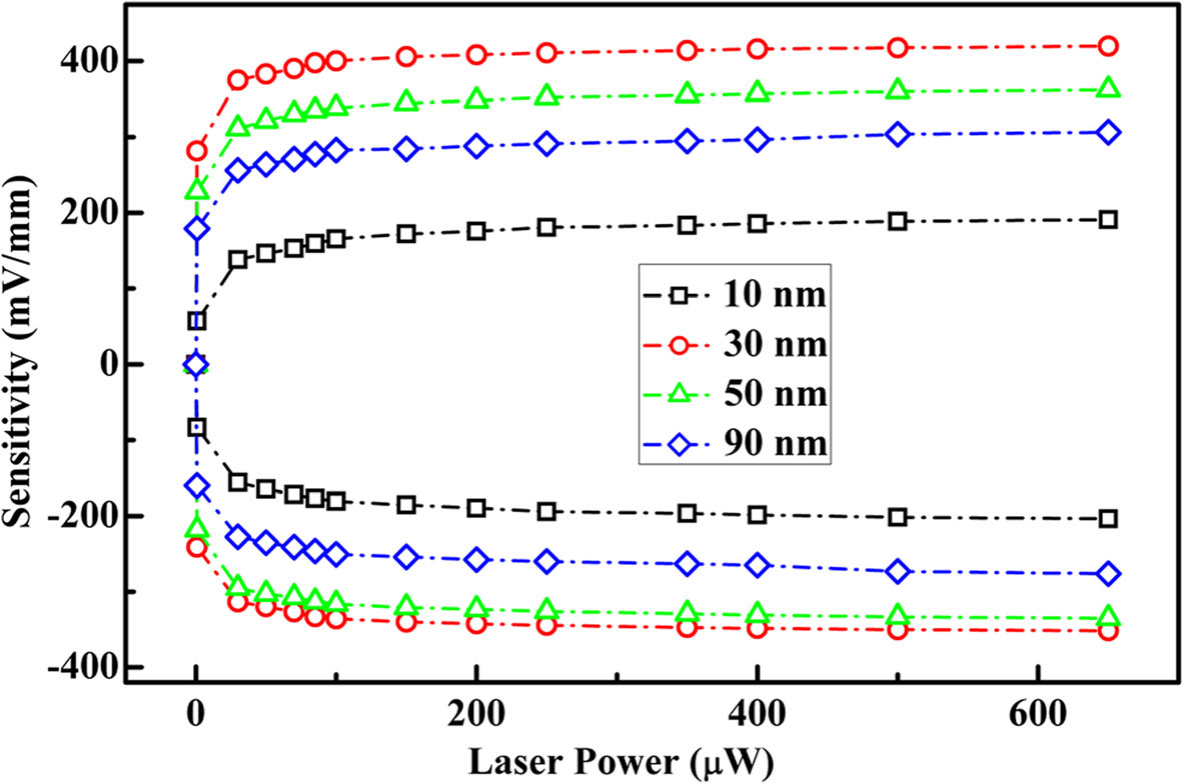
Dependence of positive and negative sensitivities of the fabricated MoS_2_/*n*-GaAs heterojunction on laser powers, respectively

Figure 5 shows the LPE sensitivity of the MoS_2_/*n*-GaAs heterojunction as a function of the laser power and the thickness (*d*_MoS2_) of the MoS_2_ film. The sensitivity is defined by *S* = LPV_max_/*L*. Obviously, the *S* increases drastically with increasing laser power initially but then slowly saturates when the power further increases. This saturation could be caused by the rapidly increasing recombination rate of the photoexcited holes with increasing laser intensity in the illuminated region [20]. As shown in the figure, an obvious LPE and a high sensitivity can be obtained even under the weak laser illumination of 100.0 μW. From the figure, a significant dependence of the sensitivity on the thickness of the MoS_2_ films can be seen. When *d*_MoS2_ = ~ 10.0 nm, *S* = 165.4 mV mm^−1^ under the laser illumination of 100.0 μW. With increasing film thickness, *S* increases gradually. When *d*_MoS2_ = 30.0 nm, *S* reaches 416.4 mV mm^−1^. This sensitivity is much larger than the reported MoS_2_/Si devices [17, 18]. After *d*_MoS2_ > 30.0 nm, *S* decreases with further increasing MoS_2_ thickness. When *d*_MoS2_ = 90.0 nm, *S =* 283.3 mV mm^−1^. Thus, to obtain the largest LPE and sensitivity, there is an optimum thickness of the MoS_2_ film in the fabricated MoS_2_/*n*-GaAs, about 30.0 nm.

**Fig. 6:**
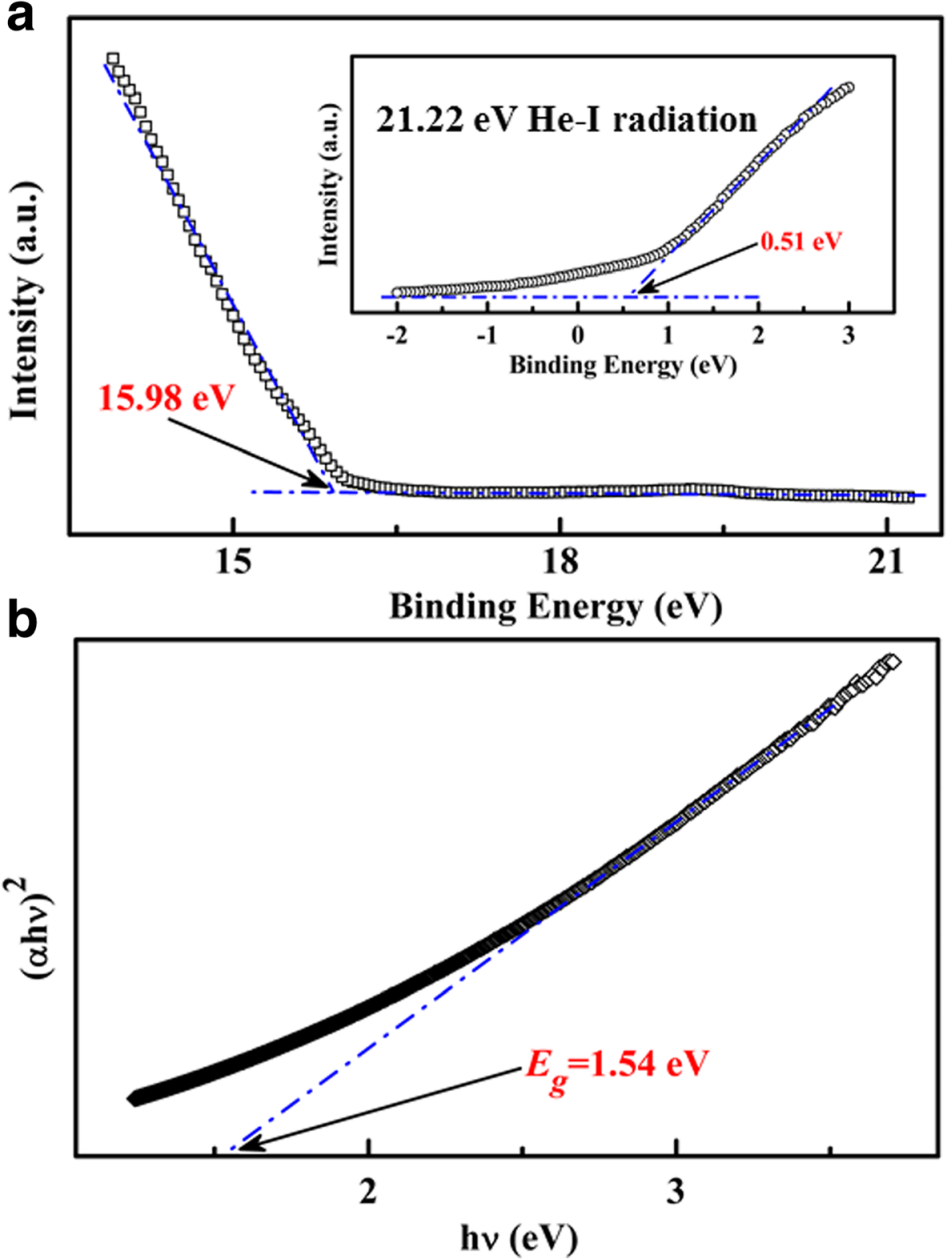
**a** UPS spectra of the MoS_2_ film on GaAs, showing the Fermi level of the films and the distance between the Fermi level and the top edge of the conduction band. **b** (*αhν*)^2^~*hν* curve from UV spectrum of the MoS_2_ film on quartz substrate under the same deposition condition

Figure 6a presents the UPS spectra of the MoS_2_ film on the Si substrate. The work function (*W*) of the film can be calculated from the difference between the cut-off of the highest binding energy and the photon energy of the exciting radiation. From the figure, *W* = 5.24 eV can be obtained. The distance (∆*E*) between the valence band (*E*_V_) and the Fermi level (*E*_F_) of MoS_2_ film can be extracted from the onset energy, as shown in the inset. The ∆*E* for the MoS_2_ film is about 0.51 eV. Using the data from the transmittance spectrum of the MoS_2_ film on quartz substrate, (*αhν*)^2^ is plotted as a function of photon energy *hν*, wherein *h* is the Planck constant and *ν* is the photon frequency. The *α* is the absorption coefficient, calculated by *αd* = ln(1/*T*) [21], wherein *d* and *T* are the thickness and transmittance of the film, respectively. The band gap (*E*_g_) of the film can be determined from the intercept of the line on the *hν* axis, *E*_g_ = 1.54 eV, as shown in Fig. 6b. Based on these energy-band parameters, the *p*-type behaviour of the MoS_2_ film can be determined, which can be further proved by Hall measurements. The Hall results show that the concentration of the hole-type carrier and the mobility are about 3.8 × 10^15^ cm^−3^ and 11.2 cm^2^ V^−1^ s^−1^, respectively.

**Fig. 7:**
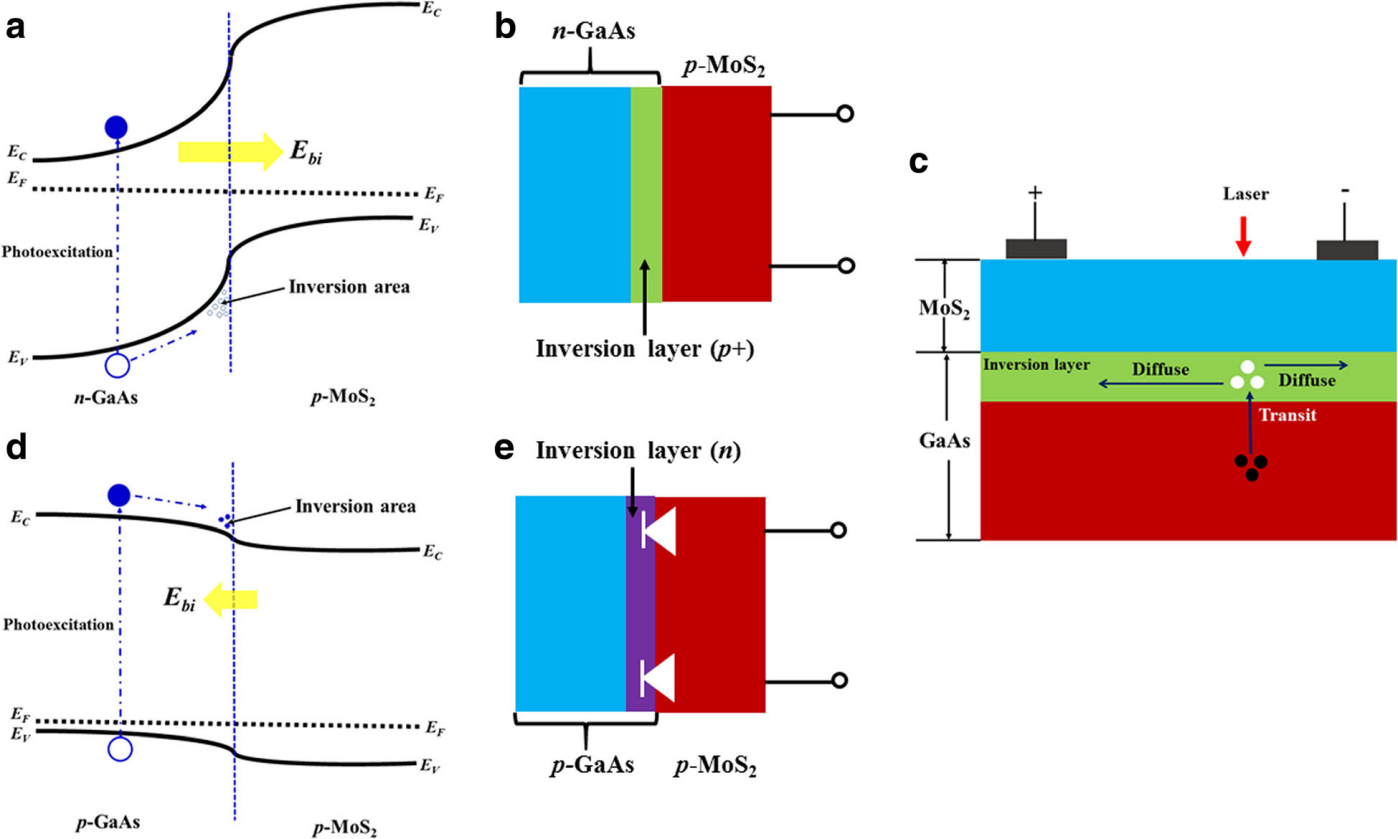
**a** Energy-band alignment of the MoS_2_/*n*-GaAs heterojunction, and **b** the corresponding illustration of the electrical contacting at the interface. **c** Mechanism of the LPE in the MoS_2_/*n*-GaAs heterojunction. **d** Energy-band alignment of the MoS_2_/*p*-GaAs heterojunction, and **e** the corresponding illustration of the electrical contacting at the interface

In order to clarify the mechanisms of the LPE in the MoS_2_/GaAs heterojunctions, the energy-band diagrams at the interface are constructed based on the results in Fig. 6. Here, *E*_g_ = 1.42 eV and *E*_F_ = 4.17 eV for *n*-GaAs are taken into account to construct the band structure [22]. When the MoS_2_ film is deposited onto the GaAs substrate, the electrons flow from the GaAs into the MoS_2_ film at the interface due to the higher *E*_F_ of the GaAs. The flowing process stops when Fermi levels are equal and the MoS_2_/GaAs *p*-*n* junction is fabricated, as shown in Fig. 7a. Consequently, a built-in field (*E*_bi_) is formed at the interface of the MoS_2_/GaAs heterojunction. *E*_bi_ = [*E*_F_(*n*-GaAs) − *E*_F_(MoS_2_)]/*e* = 1.07 V and it points from the GaAs to the MoS_2_ film. Thus, the asymmetric longitudinal *I*-*V* curve is shown in Fig. 3a. Due to the existence of a strong *E*_bi_, large quantities of hole-type carriers are accumulated near the interface and an inversion layer is formed in the *n*-GaAs substrate near the interface, as shown in Fig. 7b. Similar with the two-dimensional hole gas (2DHG) [23], the inversion layer could exhibit the feature of high conduction due to the high sheet concentration of the holes (*p+*). From Fig. 3b, we can see that the conduction of the inversion layer is much larger than the MoS_2_ film. Thus, the conduction between two electrodes on the same side of the MoS_2_ film is dominated by the inversion layer rather than the MoS_2_ film. When the junction is partially illuminated by the laser, the light is absorbed and the electron-hole pairs in the MoS_2_ film and GaAs can be induced, respectively, as shown in Fig. 7c. However, it can be expected that most of the laser is absorbed by the GaAs substrates due to its much larger thickness and smaller band gap. Under the laser illumination, the electron-hole pairs can only be excited in the illuminated region and spatially separated by the *E*_bi_. Due to the orientation of the *E*_bi_ pointing from GaAs to MoS_2_, the photoexcited holes flow towards the interface and enter into the inversion layer in the GaAs, as shown in Fig. 7c. The photoexcited holes in the inversion layer diffuse laterally away from the illuminated spot to the two electrodes. The concentration of the excited holes collected by the two electrodes is different for different distances from the illuminated spot. Thus, a large LPV is formed between the electrodes, and the LPE is observed in the heterojunction. This is in accord with the fitted results from Fig. 3b, and the LPE in the MoS2/*n*-GaAs heterojunctions mainly originates from the carrier diffusion. When the MoS_2_ film is deposited onto the *p*-GaAs substrate, a *p*-*p* heterojunction is formed, as shown in Fig. 7d. *E*_F_(*p*-GaAs) = 5.32 eV is used in the band diagram [22]. The *E*_bi_ of the *p*-*p* heterojunction can be calculated, 0.08 V, and its direction points from the film to the substrate. Due to the *E*_bi_, electron-type carriers are accumulated near the interface of the heterojunction and the inversion layer is formed. Thus, the LPE induced by the diffusion of the photoexcited electrons is obtained in the MoS_2_/GaAs *p*-*p* heterojunction, as shown in Fig. 4. However, the concentration of the accumulated carrier in the inversion layer might be lower due to the weak *V*_bi_ of only 0.08 V in the *p*-*p* heterojunction compared to the *p*-MoS_2_/*n*-GaAs junction. This increases the difficulties of the transport of the photoexcited electrons in the inversion layer. Seriously, the Schottky barriers can be formed between the *n*-type inversion layer and the *p*-MoS_2_ film, as shown in Fig. 7e. These characteristics of the *p*-MoS_2_/*p*-GaAs junction suppress the collection of the photoexcited electrons at the electrodes. As a result, the LPE could be reduced largely. As shown in Fig. 4b, the LPV_max_ for the *p*-*p* junction is only 7.3 mV while it reaches 208.2 mV in the *p*-*n* junction.

It usually happens in the reported heterojunction-type PSDs that the LPE can be tuned by changing the thickness of the cap layers [20]. This can be well understood by considering the recombination of the photoexcited carriers in the film and the evolution of the built-in field. In the MoS_2_/GaAs *p*-*n* junction, the recombination of photon-generated carriers can be enhanced due to the long transporting path in the thick MoS_2_ film before they are collected by the electrodes. This reduces the LPE of the heterojunctions. Reversely, a thinner film can greatly decrease the recombination, which causes the increase of the LPE. However, when the MoS_2_ thickness is smaller than the critical value, the *E*_bi_ at the interface decreases with further decreasing MoS_2_ thickness [24]. This can reduce the separation of photon-generated electron-hole pairs, and the LPE decreases. Thus, there is an optimum thickness of the MoS_2_ film for obtaining the highest LPE, about 30 nm.

## Conclusions

In summary, MoS_2_ thin films were deposited on the surface of the GaAs substrate via magnetron sputtering technique. A large LPE was obtained in the fabricated MoS_2_/*n*-GaAs heterojunction, and the dependence of the LPV on the position of the laser illumination showed good linearity. Due to the formation of the strong built-in field at the interface, the MoS_2_/*n*-GaAs heterojunction exhibited a high sensitivity of 416.4 mV mm^−1^, while the value was only 7.3 mV mm^−1^ for the MoS_2_/*p*-GaAs. Our results further showed that the LPE exhibited obvious dependence on the thickness of the MoS_2_ films and about 30.0 nm was the optimum thickness of the MoS_2_ film to acquire the highest LPE in the fabricated MoS_2_/*n*-GaAs heterojunctions. The mechanisms to the LPE in the MoS_2_/GaAs devices were clarified based on the energy-band alignment at the interface.

## Additional file

Additional file 1: Large lateral photovoltaic effect in MoS_2_/GaAs heterojunction. Figure S1. Cross-sectional SEM images of the as-grown MoS_2_ film. (DOCX 731 kb)

## Abbreviations

∆*E*: Distance between *E*_V_ and *E*_F_
*d*_MoS2_: Thickness of the MoS_2_ film
*E*_bi_: Built-in field
*E*_C_: Conduction band level
*E*_F_: Fermi energy level
*E*_g_: Energy-band gap
*E*_V_: Valence band level
*I*-*V*: Current-voltage
LPE: Lateral photovoltaic effect
LPV: Lateral photovoltage
LPV_max_: Maximum lateral photovoltage
MoS_2_: Molybdenum disulfide
PSD: Position-sensitive detector
UPS: Ultraviolet photoelectron spectroscopy
*W*: Work function
