# Supplementary material for: Large Lateral Photovoltaic Effect in MoS2/GaAs Heterojunction
Source: Nanoscale Res Lett. 2017 Oct 10;12:562. doi: 10.1186/s11671-017-2334-z (PMC5635143; doi:10.1186/s11671-017-2334-z)
Supplement: Additional file 1: — Large lateral photovoltaic effect in MoS2/GaAs heterojunction. Figure S1. Cross-sectional SEM images of the as-grown MoS2 film. (DOCX 731 kb) [file 11671_2017_2334_MOESM1_ESM.docx]

**Additional file 1**

**Large lateral photovoltaic effect in MoS_2_/GaAs heterojunction**


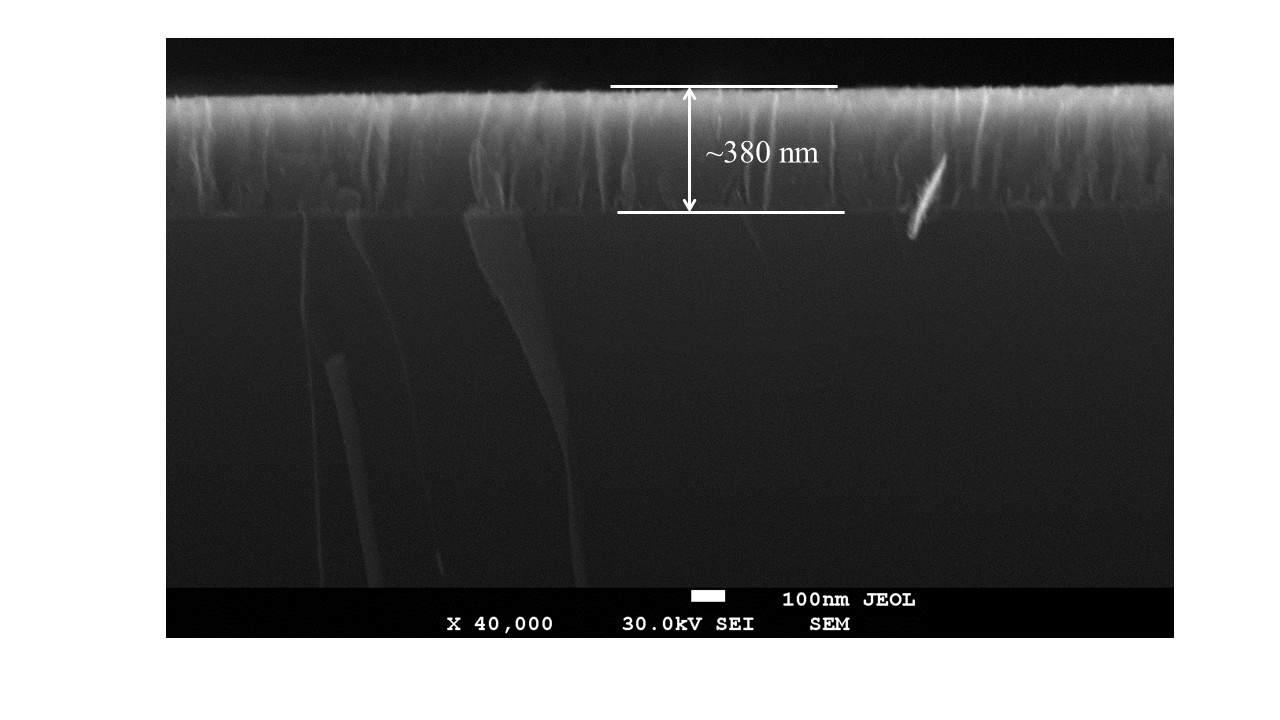


Figure S1. Cross-sectional SEM images of the as-grown MoS_2_ film.
